# Treatment of post-meniscectomy knee symptoms with medial meniscus replacement results in greater pain reduction and functional improvement than non-surgical care

**DOI:** 10.1007/s00167-021-06573-0

**Published:** 2021-04-21

**Authors:** Kenneth R. Zaslav, Jack Farr, Richard Alfred, R. Maxwell Alley, Michael Dyle, Andreas H. Gomoll, Christian Lattermann, Brian P. McKeon, Christopher C. Kaeding, Thomas Giel, Elliott B. Hershman

**Affiliations:** 1 Ortho Virginia, Virginia Commonwealth University, 7858 Shrader Road, Richmond, VA 23294 USA; 2 OrthoIndy, Greenwood, IN USA; 3 The Bone and Joint Center, Albany, NY USA; 4 Telos Partners, LLC, Denver, CO USA; 5 Hospital for Special Surgery, New York, NY USA; 6 Brigham and Women’s Faulkner Hospital Orthopaedic Center, Boston, MA USA; 7 Boston Sports and Shoulder Center, Waltham, MA USA; 8 The Ohio State University Wexner Medical Center Sports Medicine, Columbus, OH USA; 9 OrthoSouth, Memphis, TN USA; 10 Northwell Health Physician Partners, Orthopaedic Institute at Lenox Hill, New York, NY USA

**Keywords:** Partial meniscectomy, Medial meniscus, Meniscus lesion, Meniscus tear, Implant, Replacement, NUsurface, Non-surgical care, RCT, KOOS

## Abstract

**Purpose:**

Partial meniscectomy is a common orthopedic procedure intended to improve knee pain and function in patients with irreparable meniscal tears. However, 6–25% of partial meniscectomy patients experience persistent knee pain after surgery. In this randomized controlled trial (RCT) involving subjects with knee pain following partial meniscectomy, it was hypothesized that treatment with a synthetic medial meniscus replacement (MMR) implant provides significantly greater improvements in knee pain and function compared to non-surgical care alone.

**Methods:**

In this prospective, multicenter RCT, subjects with persistent knee pain following one or more previous partial meniscectomies were randomized to receive either MMR or non-surgical care. This analysis evaluated the 1-year outcomes of this 2-year clinical trial. Patient-reported knee pain, function, and quality of life were measured using nine separate patient-reported outcomes. The primary outcomes were the pain subscale of the Knee injury and Osteoarthritis Outcome Score (KOOS) and the average of all five KOOS subscales (KOOS Overall). Treatment cessation was defined as permanent device removal in the MMR group and any surgical procedure to the index knee in the non-surgical care group.

**Results:**

Treated subjects had a median age of 52 years old (range 30–69 years) and one or more previous partial meniscectomies at a median of 34 months (range 5–430 months) before trial entry. Among 127 subjects treated with either MMR (*n* = 61) or non-surgical care (*n* = 66), 11 withdrew from the trial or were lost to follow-up (MMR, *n* = 0; non-surgical care, *n* = 11). The magnitude of improvement from baseline to 1 year was significantly greater in subjects who received MMR in both primary outcomes of KOOS Pain (*P* = 0.013) and KOOS Overall (*P* = 0.027). Treatment cessation was reported in 14.5% of non-surgical care subjects and only 4.9% of MMR subjects (n.s.).

**Conclusion:**

Treatment with the synthetic MMR implant resulted in significantly greater improvements in knee pain, function, and quality of life at 1 year of follow-up compared to treatment with non-surgical care alone.

**Level of evidence:**

I.

**Supplementary Information:**

The online version contains supplementary material available at 10.1007/s00167-021-06573-0.

## Introduction

The meniscus plays vital protective roles in the knee, which include supporting joint stability as well as absorption and distribution of loads that are generated during normal weight-bearing and gait [3]. Depending on their size and location, meniscal tears can disrupt these protective functions, resulting in altered joint biomechanics and increased contact pressure on the articular cartilage and bone, with pain and disability as common outcomes [9, 23, 26]. A 2019 systematic review and meta-analysis of asymptomatic, uninjured knees reported that a meniscal tear was observed via magnetic resonance imaging (MRI) in 4% of individuals under 40 years of age and 19% of individuals at least 40 years of age [8]. Among patients with knee pain, the incidence of meniscal tears on MRI has been reported to exceed 70% [10]. Multiple studies have shown that torn or dysfunctional menisci may be associated with impaired mobility, loss of articular cartilage, and the development of radiographic knee osteoarthritis [11, 14, 18, 28]. Thus, maintaining meniscal function is critical to supporting mobility and the long-term health of the knee.

Partial meniscectomy is the most common surgical treatment for symptomatic, irreparable meniscus tears, with 760,000 outpatient procedures performed annually in the US [17]. The principal goals of partial meniscectomy are to relieve knee pain and functional limitations through the surgical removal of loose, unstable meniscal fragments and smoothing of frayed edges to prevent additional tearing. Evidence from randomized controlled trials (RCT) has suggested that partial meniscectomy may not be superior to non-surgical care alone or sham surgery, but some trials had important study design limitations and high cross-over rates [1, 7, 27, 32, 33, 35, 36]. Nonetheless, expert consensus statements and professional societies recommend partial meniscectomy for patients with irreparable meniscus tears and mechanical knee symptoms that are refractory to at least 3 months of non-surgical care [5, 19]. For these patients, partial meniscectomy is safe, with serious complications occurring in less than 1% of cases [2].

Clinical studies have shown that 6–25% of patients who previously received a partial meniscectomy experience persistent or recurrent pain within 1–2 years after surgery [20, 33, 37]. Moreover, arthroscopic partial meniscectomy is associated with a significantly increased risk of radiographic osteoarthritis following surgery [24, 29]. Current treatment options for patients with knee pain and impaired mechanical function following partial meniscectomy are limited. In addition, there are no widely accepted clinical practice guidelines that result in relief for these patients. Therefore, there is a critical medical need for effective treatment options that relieve persistent pain, restore meniscal function, and potentially slow the long-term risk of degenerative knee conditions in patients with knee pain following a partial meniscectomy, but who are not clinically ready or indicated for joint replacement.

A synthetic medial meniscus implant (NUsurface^®^, Active Implants LLC, Memphis, TN) was designed to replace previously resected meniscal tissue and provide relief from knee pain and impaired function in patients with knee symptoms following partial meniscectomy. The medial meniscus replacement (MMR) implant was designed to mimic the biomechanical function of the natural medial meniscus, is composed of a pliable yet durable polycarbonate-urethane (PCU) polymer with embedded ultrahigh molecular weight polyethylene (UHMWPE) reinforcement fibers, and does not require fixation to bone or soft tissue [31]. Previous studies have shown that the MMR implant mimics the motion of the native meniscus in the knee joint during flexion, restores pressure distribution and force transmission in the meniscectomized knee to near normal levels, and maintains normal knee kinematics and joint space [31]. This multicenter RCT was designed to test the hypothesis that placement of the MMR implant is a superior treatment strategy compared to non-surgical care for patients with knee pain following one or more previous partial meniscectomies. This study reports the 1-year midpoint results from this 2-year clinical trial.

## Materials and methods

VENUS (Verifying the Effectiveness of the NUsurface^®^ System, NCT02108496) is a prospective, multicenter, superiority, randomized controlled trial. Prior to patient recruitment, the trial protocol was approved by the Institutional Review Board at each trial site. All subjects gave written informed consent prior to enrollment. The trial was conducted in accordance with the principles of the Declaration of Helsinki and good clinical practice.

Eligible patients were randomized in a 1:1 ratio to receive either the investigational MMR implant or non-surgical care. Randomization was performed using a centralized, web-based, block-randomization system that enabled random treatment assignment from a secure web portal at each study site. Subjects were enrolled at 11 sites in the US from 2014 to 2018. Trial sites were monitored periodically to confirm that written informed consent was obtained for all participants and to ensure adherence to the trial plan, follow-up schedules, accurate recording of clinical data, and 100% source verification of the study data. No analyses of the outcome data occurred before subject enrollment were complete.

### Participants

All subjects underwent a complete medical examination and MRI of the index knee before participation. Eligible subjects were between 30 and 75 years of age, had knee pain (≤ 75 on a 100-point scale, where 100 is “no pain”), had a medial partial meniscectomy at least 6 months prior to evaluation as confirmed by patient history and MRI, had neutral alignment within 5° of the mechanical axis, had at least 2 mm of intact medial meniscal rim, understood the English language, were willing to be randomized into either arm of the trial, and were able to complete trial follow-up visits. Patients were excluded if they had any of the following: evidence of Outerbridge Grade IV cartilage loss on the medial tibial plateau or medial femoral condyle, Outerbridge Grade III or IV cartilage loss in the lateral compartment, detachment of the posterior medial meniscal root, > 5° of varus or valgus knee deformity, an anterior cruciate ligament reconstruction surgery within the previous 9 months, or body mass index (BMI) exceeding 32.5 kg/m^2^. A full list of the inclusion and exclusion criteria are published with the trial registration (ClinicalTrials.gov: NCT02108496).

### Treatment groups

Non-surgical care treatments were prescribed upon randomization. A specific treatment algorithm was not defined in the protocol. Rather, surgeons followed their standard clinical practice for the treatment of post-partial meniscectomy knee pain and tailored the non-surgical care to address the needs and symptoms of each subject. Over time, non-surgical care could be modified to meet changing needs and symptoms. The non-surgical treatment options included intra-articular injections of hyaluronic acid and/or corticosteroids; prescription and/or non-prescription oral medications; physical therapy, low-impact aerobic exercise, and/or strength training; ice and/or heat therapy; unloader knee braces, compression sleeves, crutches and/or canes; weight loss; activity restrictions; and/or shoe inserts or other orthotic devices. The following options were not allowed in the non-surgical care arm: platelet-rich plasma injections, acupuncture, other devices that do not have regulatory approval, and all surgical treatments on index knees.

**Fig. 1:**
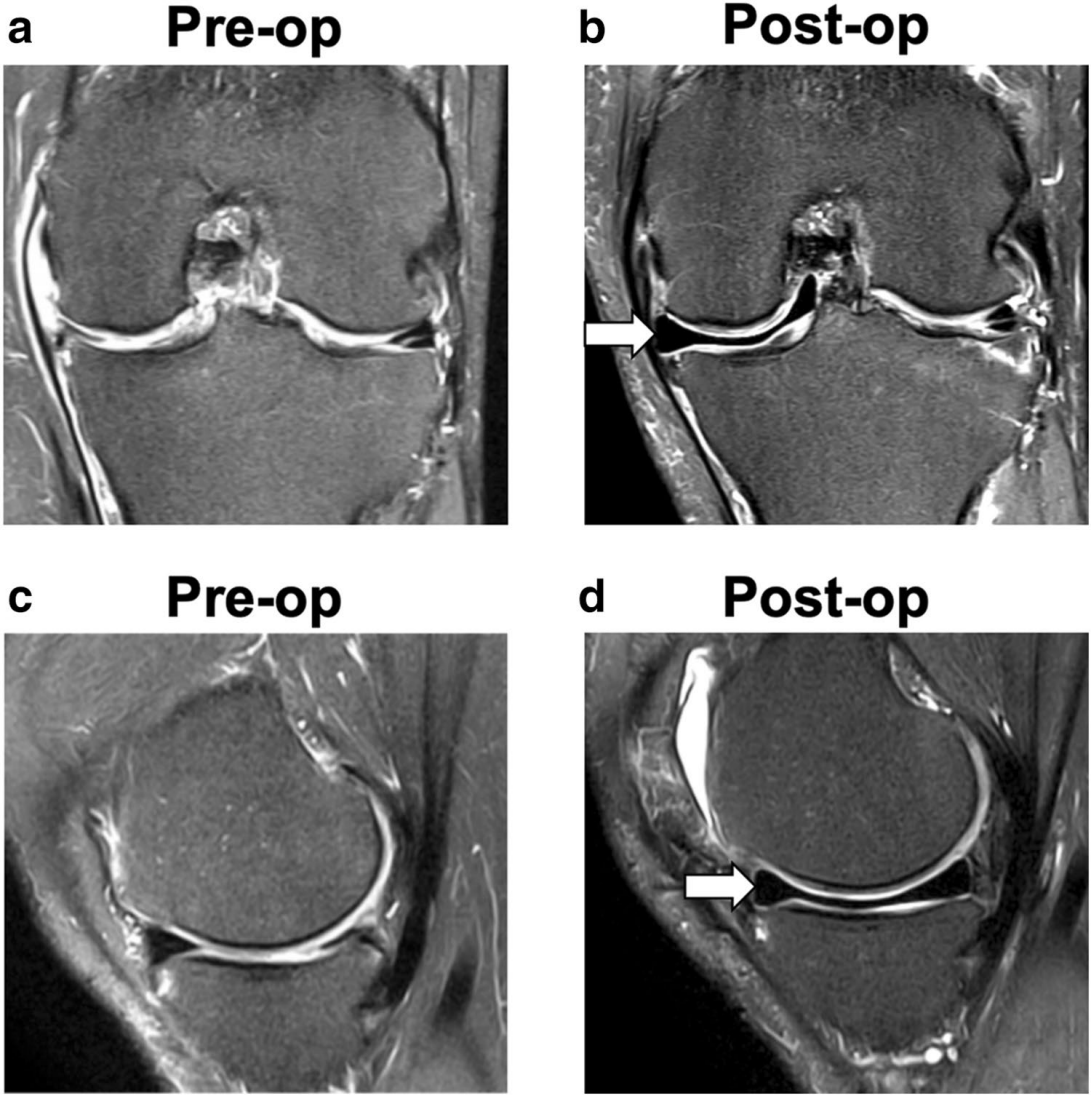
Magnetic resonance imaging (MRI) of a knee before (pre-op; **a** and **c**) and after (post-op; **b** and **d**) implantation of the MMR implant from the coronal (**a**, **b**) and sagittal (**c**, **d**) views. Arrows indicate the MMR implant

For the surgical arm, the MMR implant was used. The synthetic MMR implant (NUsurface^®^ Meniscus Implant; Active Implants LLC; Memphis, TN) is made from a durable polycarbonate-urethane polymer (Bionate^®^ 80A PCU; DSM Biomedical; Exton, PA) and contains circumferential reinforcement fibers composed of ultrahigh molecular weight polyethylene (UHMWPE; Dyneema Purity^®^ Fibers; DSM Biomedical; Exton, PA). The MMR implant replaces meniscal tissue in the medial compartment (Fig. 1) and does not require fixation to soft tissue or bone. The surgical procedure involves an arthroscopic sub-total meniscectomy of the medial meniscus, leaving a vertical 2–4 mm meniscal rim, and an arthrotomy of 4–8 cm to complete the joint preparation and insert the meniscus implant. The MMR implant is available in seven sizes; the target size for each patient is selected preoperatively based on MRI and confirmed intraoperatively using a radiopaque trial implant and fluoroscopy prior to placement of the definitive implant.

Immediate post-operative rehabilitation included compression bandage, knee immobilization for ambulation with weight-bearing as tolerated, and range-of-motion exercises 3–4 times/day. Approximately 1–2 weeks after surgery, patients discontinued knee immobilization with full weight bearing as tolerated, and quadriceps strengthening exercises. By 2–6 weeks after surgery, cycling, straight leg, and closed chain exercises were permitted with full range of motion. After 6 weeks, low-impact activities and open chain exercises were permitted. Subjects in both treatment groups were recommended to not engage in higher impact activities such as contact sports, running/jogging, soccer, or skiing, as a few examples.

### Follow-up and outcome measures

Subjects completed clinical follow-up visits at 1.5 months, 6 months, and 1 year after treatment initiation; subjects will continue follow-up through at least 2 years. The primary trial endpoint was defined as a composite overall success measure at 2 years of follow-up. Overall success was defined as improvement in the Knee injury and Osteoarthritis Outcome Score (KOOS) subscales of KOOS Pain and KOOS Overall, no MMR implant removal, and an MRI confirming that the MMR implant is in one piece and not subluxed. Overall success will be assessed for superiority at the 2-year primary endpoint. In this 1-year midpoint analysis, improvement in knee-specific pain, function, and quality of life were assessed using the 5 KOOS subscales of pain, symptoms, activities of daily living (ADL), sports and recreation, and quality of life (QOL) as well as the average of the five subscales (KOOS Overall) [30]. KOOS Pain and KOOS Overall were the primary outcome measures, as they will contribute to the primary trial endpoint of overall success. Responders were calculated for each KOOS domain and were defined as subjects who achieved a ≥ 20-point improvement from baseline to 1 year of follow-up, or a score at 1 year exceeding thresholds defined for a symptomatic knee [15]. A 10-point improvement in KOOS Pain represents the minimum change that is required to detect a clinically significant improvement from the patient’s perspective [22].

Additional validated patient-reported outcome measures included the Visual Analog Scale (VAS) for leg pain, the Western Ontario Meniscal Evaluation Tool (WOMET), and the International Knee Documentation Committee (IKDC) score [13].

Following randomization, subjects were not permitted to cross-over between treatment groups; however, subjects were permitted to cease treatment with non-surgical care or the MMR implant and receive alternative treatments after withdrawing from the clinical trial. Treatment cessation was defined as any subject who decided to discontinue the allocated per-protocol treatment to undergo a surgical procedure on the index knee (non-surgical care group) or undergo permanent removal of the MMR implant (MMR group). Following treatment cessation, patient-reported outcomes were not collected, because they no longer represented the allocated per-protocol treatment (i.e. non-surgical care or retained MMR implant). However, in the MMR group, subjects were permitted to remain in the study and undergo subsequent surgical procedures on the index knee to exchange or reposition the device, as well as other surgeries, as long as the MMR implant was not permanently removed. For MMR subjects who underwent subsequent surgical procedures on index knees, patient-reported outcomes were collected after the subsequent surgery and included in the data analysis.

### Statistical analysis

Baseline characteristics were compared between the MMR and non-surgical care groups using two-sample two-sided Student’s *t* tests, two-sided Mann–Whitney test, Fisher’s exact test, or Chi-square test. Within-group and between-group comparisons of KOOS, VAS, WOMET, and IKDC scores from baseline to each follow-up timepoint were analyzed using a repeated measures mixed-effects model plus Sidak’s multiple comparisons test. The repeated measures mixed-effects model accommodates missing values by accounting for within-subject correlation resulting from multiple measures within individual subjects. Sidak’s test was used to adjust for the multiple comparisons made between treatment groups across time points. The times from treatment initiation to treatment cessation were compared between groups using a two-sided Student’s *t* test. The rates of KOOS responders and treatment cessation were compared between groups using Fisher’s exact test. The D’Agostino and Pearson normality test was used to determine if data satisfied assumptions of normality. A two-sided *P* value of ≤ 0.05 was considered statistically significant. The required sample size for this trial was calculated to be 52 subjects per group based on assumptions of 80% power and a type I error rate of 5% to detect a statistically significant difference in a binary outcome of overall treatment success using a Chi-Square test of two proportions. Assuming a loss to follow-up of up to 15%, enrollment targets were at least 62 subjects per group.

## Results

**Fig. 2:**
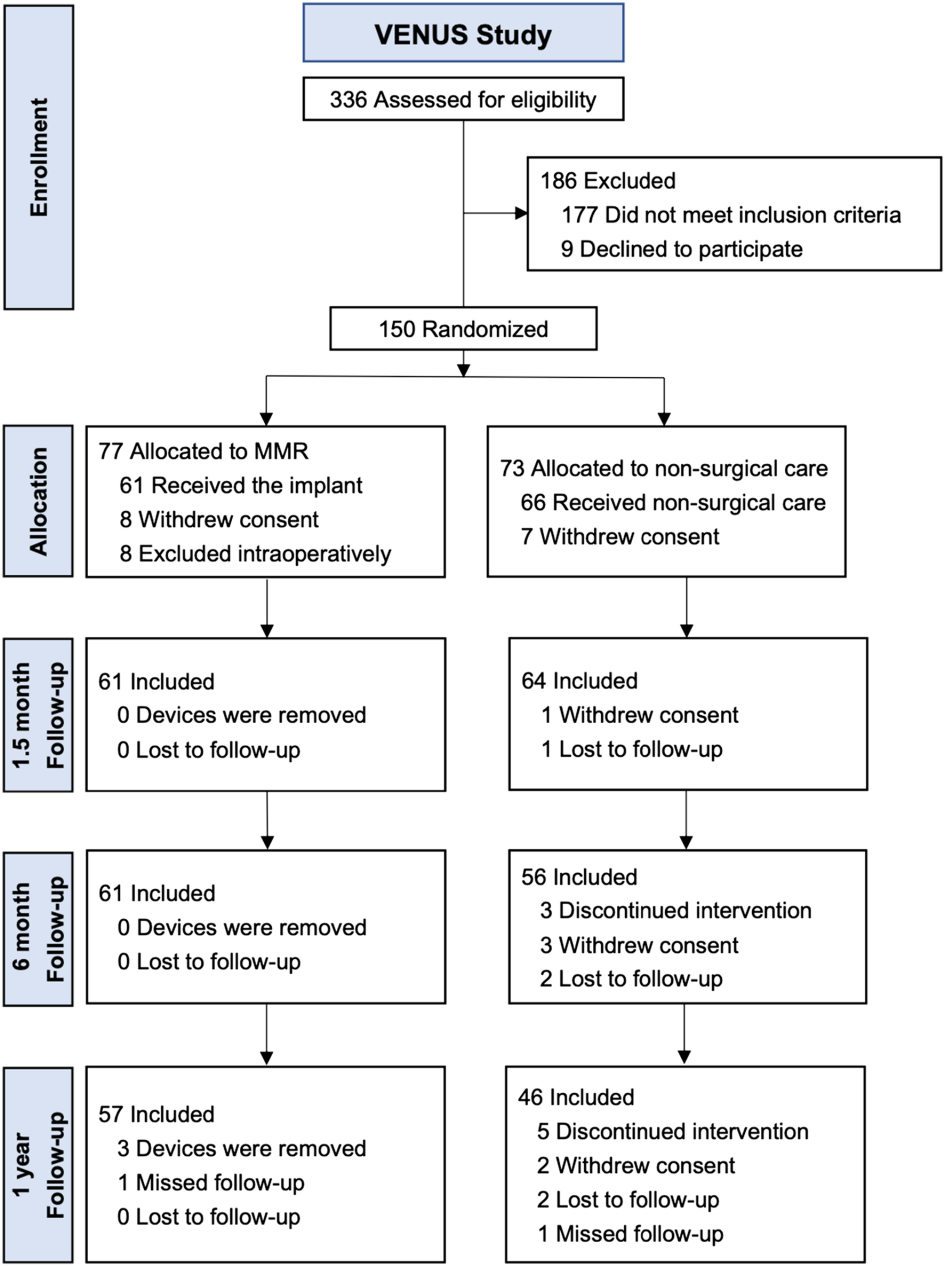
CONSORT flow diagram of patient allocation and follow-up

One hundred twenty-seven subjects were randomized and received either surgical (MMR) treatment (*n* = 61) or non-surgical care (*n* = 66; Fig. 2). The baseline patient characteristics were similar between the MMR and non-surgical care groups (Table 1). Subjects were an average of 50 years old, mostly (76%) men, and had an average BMI of 26.8 kg/m^2^. Seventy percent of subjects had one previous partial meniscectomy and 30% had two or more previous partial meniscectomies.

**Table 1:** Patient baseline characteristics

Characteristic	MMR (*n* = 61)	Non-surgical (*n* = 66)	*P* value
Age	51.0 ± 11.2	49.8 ± 10.3	n.s
Body mass index (BMI)	26.8 ± 3.2	26.8 ± 3.6	n.s
Male Gender—*n* (%)	48 (78.7%)	48 (72.7%)	n.s
Left index knee—*n* (%)	31 (50.8%)	31 (47.0%)	n.s
Number of previous partial meniscectomies			n.s
One—*n* (%)	42 (68.9%)	46 (69.7%)	
Two—*n* (%)	11 (18.0%)	14 (21.2%)	
Three or more—*n* (%)	8 (13.1%)	6 (9.1%)	
Median (range) months since last meniscectomy	34 (7–313)	35 (5–425)	n.s
KOOS			
Pain	52.1 ± 11.2	54.2 ± 15.6	n.s
Symptoms	59.4 ± 15.3	62.6 ± 16.5	n.s
ADL	63.4 ± 16.0	65.3 ± 19.9	n.s
Sports and recreation	35.5 ± 22.2	39.3 ± 21.7	n.s
QOL	27.6 ± 16.9	30.0 ± 13.5	n.s
Overall	47.6 ± 12.6	50.3 ± 14.3	n.s
VAS pain	53.3 ± 20.5	51.0 ± 23.7	n.s
WOMET	36.3 ± 16.2	39.1 ± 16.6	n.s
IKDC	42.4 ± 13.2	45.4 ± 13.9	n.s

Values are mean ± standard deviation or number (*n*) and percent (%) unless otherwise specified

*n.s.* not significant

Non-surgical treatment was designed to mimic the surgeon’s current standard non-operative treatment for persistent post-partial meniscectomy knee pain, was individually tailored to each subject, and varied from surgeon to surgeon (Table 2). The average number of hyaluronic acid injections and corticosteroid injections among subjects who received them was 3.1 (median: 3; range: 1–6) and 1.3 (median: 1; range: 1–3), respectively.

**Table 2:** Prescribed non-surgical treatments

	Intervention no. (%)
Intra-articular hyaluronic acid injection	32 (48%)
Physical therapy, non-weight-bearing or weight-bearing exercises	30 (45%)
Compression sleeves or unloader braces	29 (44%)
Prescription or non-prescription NSAIDs	23 (35%)
Activity limitation	22 (33%)
Ice or heat therapy	21 (32%)
Intra-articular corticosteroid injection	8 (12%)
Body weight reduction	5 (8%)
Non-prescription drugs, creams, vitamins, or supplements	4 (6%)
Shoe orthotic devices	3 (5%)

Values do not sum to 100% because many subjects were prescribed multiple types of non-surgical care treatments

### KOOS

**Fig. 3:**
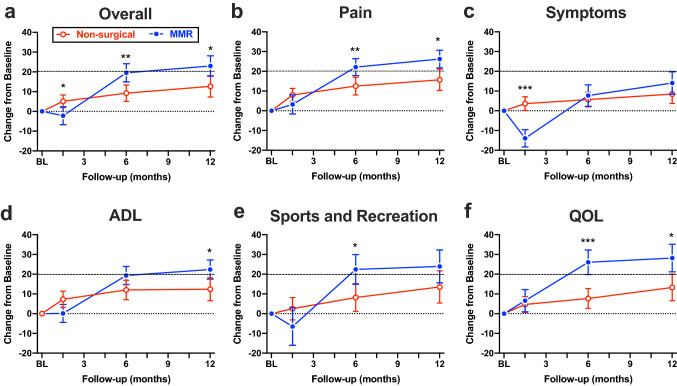
Change in KOOS subscales. Magnitude of change from baseline (BL) to 1 year of follow-up in KOOS Overall (**a**), pain (**b**), symptoms (**c**), activities of daily living (ADL; **d**), sports and recreation (**e**), and quality of life (QOL; **f**). Symbols indicate the mean and error bars are 95% confidence intervals. Open red symbols represent the non-surgical care group and closed blue symbols represent the medial meniscus replacement (MMR) implant group. Higher values indicate better improvement in pain, symptoms, function, or quality of life. *Indicates *P* < 0.05, ** indicates *P* < 0.01, and *** indicates *P* < 0.001 for MMR group vs non-surgical care group

**Fig. 4:**
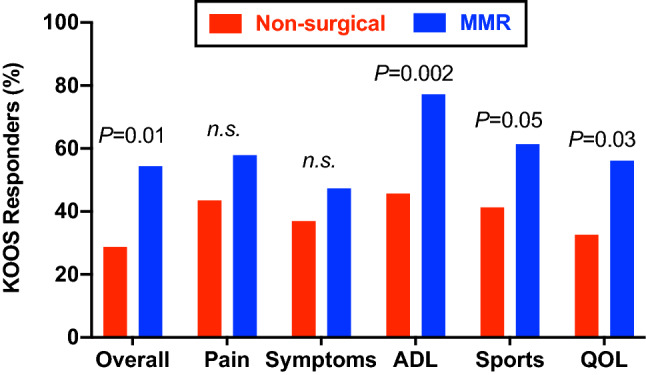
Percentage of responders in each KOOS subscale. The percentage of subjects who achieved a ≥ 20-point improvement in each KOOS subscale at 1 year, or a 1-year score exceeding thresholds defined for a symptomatic knee. Red bars represent the non-surgical care group and blue bars represent the medial meniscus replacement (MMR) group. *n.s.* not significant

Both surgical and control groups showed improvement in KOOS scores at 1 year compared with baseline (*P* < 0.05). The magnitude of improvement from baseline to 1 year of follow-up was significantly greater in the MMR group compared to non-surgical care for the primary outcomes of KOOS Overall (Fig. 3a; *P* = 0.027) and KOOS Pain (Fig. 3b; *P* = 0.013). Improvements were also significantly greater in the MMR group for KOOS ADL and QOL, but the differences were not statistically significant for KOOS Symptoms or Sports and Recreation (Fig. 3c–f). The KOOS responder rate was significantly higher in the MMR group for KOOS Overall, ADL, Sports and Recreation, and QOL (Fig. 4).

### VAS pain, WOMET, and IKDC

VAS pain and WOMET scores were significantly better among MMR subjects compared to the non-surgical care group at 6 months and 1 year. Specifically, the magnitude of improvement from baseline to 1 year was significantly greater in the MMR group vs non-surgical care for VAS Pain (*P* = 0.004), WOMET (*P* = 0.002) and IKDC scores (*P* = 0.03) compared to non-surgical care (Additional File 1).

### Treatment cessation

There was a higher frequency of treatment cessation in the non-surgical care group (8/55; 14.5%) compared to the MMR group (3/61; 4.9%), but the difference was not statistically significant. Among control subjects, treatment cessation was associated with a variety of arthroscopic procedures, high tibial osteotomy, or unicondylar knee replacement; the three MMR subjects ceased treatment following device rotation or subluxation (Table 3).

**Table 3:** Surgical procedures associated with treatment cessation

Type of surgery	Unique subjects *n* (%)
MMR group	3 (4.9%)
Permanent implant removal following device rotation	2 (3.3%)
Permanent implant removal following device dislocation	1 (1.6%)
Non-surgical care group	8 (14.5%)
Exploratory arthroscopy	1 (1.8%)
Medial and lateral partial meniscectomy	1 (1.8%)
Medial arthroscopic partial meniscectomy, synovectomy, and chondroplasty	1 (1.8%)
Medial partial meniscectomy and posterolateral corner reconstruction	1 (1.8%)
High tibial osteotomy	1 (1.8%)
Trochlear osteochondral allograft transplantation	1 (1.8%)
Meniscus transplantation	1 (1.8%)
Unicondylar knee replacement	1 (1.8%)

### Subsequent surgical procedures

In addition to the treatment cessations described above, there were eight subsequent surgical procedures in seven MMR subjects (11.5%) over 1 year of follow-up (Table 4). One subject had two subsequent surgical procedures during the 1st year to reposition the implant after it rotated as a result of traumatic twisting of the knee. Following this second incident, the implant was exchanged with a larger size to avoid instability. In addition, two more subjects underwent a device exchange procedure due to implant damage and dislocation during exercise or sports.

**Table 4:** Summary of subsequent surgical procedures on index knees in the MMR group

Type of subsequent surgery	Events *n*	Unique subjects *n* (%)
Permanent implant removal	3	3 (4.9%)
Rotation	2	2 (3.3%)
Dislocation	1	1 (1.6%)
Implant exchange	3	3 (4.9%)
Damage + Dislocation	2	2 (3.3%)
Rotation	1	1 (1.6%)
Implant repositioned	1	1 (1.6%)
Rotation	1	1 (1.6%)
Other^a^	4	4 (6.6%)

^a^ Other subsequent surgical procedures included synovectomy, chondroplasty, notchplasty, debridement, or adhesion lysis

Four MMR subjects underwent other subsequent surgical procedures that did not involve repositioning or exchange of the MMR implant. The implant was confirmed to be intact and in a stable position in these cases. One subject underwent a synovectomy, chondroplasty, and notchplasty with removal of osteophytes for recurrent effusion. Three subjects underwent lysis of adhesion or notchplasty to address patient-reported difficultly with full knee flexion.

## Discussion

The most important finding of the present study was the MMR implant resulted in significantly greater improvements in the primary outcome measures of KOOS Pain and KOOS Overall at 1 year of follow-up compared to standard non-surgical care in patients with persistent knee pain following previous partial meniscectomy. Furthermore, a greater proportion of subjects in the non-surgical care group discontinued treatment and underwent a subsequent surgical procedure compared to the proportion of subjects in the MMR group who had the device permanently removed; however, the difference was not statistically significant. These findings are of important clinical relevance considering the highly limited treatment options for this patient population. These results may help to inform patients and healthcare providers regarding treatment decisions and to set expectations for the 1st year of treatment.

Currently, there are limited treatment options for patients with knee pain following partial meniscectomy. Intra-articular corticosteroid injections not only may provide temporary (< 6 months) relief from pain, but also can exacerbate the loss of articular cartilage [25]. Hyaluronic acid injections provide transient relief from pain, but require frequent recurrent injections and may not provide a clinically meaningful benefit immediately after partial meniscectomy [6, 12]. Meniscal allograft transplantation is associated with significant improvements in knee pain and function in younger patients (mean age: 28 years; range 17–46 years) with symptomatic post-meniscectomy knees [34]. However, allograft transplantation is limited to younger patients (< 50 years), by the supply of tissue bank allografts, and can be challenging to match in size and shape to ensure proper fit [16, 21]. Finally, treatments such as unicompartmental or total knee replacement are intended for patients over 60 years old and those with end-stage degenerative knee conditions, because the risk of revision surgery is approximately 20–35% for patients aged 50–54 years [4]. In this study, the MMR implant appears to provide encouraging outcomes at the 1-year midpoint of a 2-year superiority trial, which may indicate that this will be an important treatment option to bridge the current treatment gap for patients with persistent knee pain after partial medial meniscectomy.

There are three notable advantages of the MMR implant. First, the implant does not require fixation or anchoring to bone or soft tissue, which allows the implant to translate through the full range of motion during knee articulation. Of course, the lack of fixation may allow for rotation or subluxation of the implant, and also allows the implant to be readily exchanged or repositioned during a subsequent surgery if required. Second, the pliable, PCU-based MMR implant mimics the biomechanical function of the native meniscus, conforms to joint surfaces to distribute loads, and has been shown to slow the onset of articular cartilage degeneration in an animal model of meniscectomy [31, 38]. With similar biomechanical properties to the native meniscus, mechanical wear and failure of the implant could occur under excessive or abnormal use. Finally, the surgical procedure does not require extensive resection of bone or alteration of the anatomy, allowing for future conversion to a reconstructive surgical procedure, such as a joint replacement, when determined clinically necessary. Therefore, the MMR implant can serve as a bridging treatment by delaying the time between partial meniscectomy and more invasive procedures such as knee replacement.

This trial has several important strengths, including enrollment of patients across 11 trial sites in the United States, strict patient eligibility criteria, and randomized treatment allocation. However, one limitation of the trial is that 16.7% of subjects in the non-surgical care group were lost to follow-up or withdrew within the 1st year. In addition, subject blinding was not possible due to the nature of the treatments, which may have exposed the results to potential expectation bias during the collection of patient-reported outcomes. Finally, functional assessments focused on patient-reported outcomes and did not include objective measurements, such as range of motion, because patient perception information is an important indicator for determining the clinical relevance and impact of a new therapy.

## Conclusions

Subjects with knee pain following partial meniscectomy who were randomly assigned to receive MMR had significantly greater improvements from baseline in knee pain, activities of daily living, and quality of life at 1 year of follow-up of an ongoing 2-year clinical trial compared to non-surgical care alone. Differences in mechanical symptoms and sports and recreation were not statistically significant between the groups at 1 year.

## Supplementary Information

Below is the link to the electronic supplementary material.

Supplementary file1 (DOCX 20 KB)

## Abbreviations

MMR: Medial meniscus replacement
VENUS: Verifying the Effectiveness of the NUsurface^®^ System
RCT: Randomized controlled trial
BMI: Body mass index
MRI: Magnetic resonance imaging
KOOS: Knee Injury and Osteoarthritis Outcome Score
ADL: Activities of daily living
QOL: Quality of life
VAS: Visual Analog Scale
WOMET: Western Ontario Meniscal Evaluation Tool
IKDC: International Knee Documentation Committee
PCU: Polycarbonate-urethane
UHMWPE: Ultrahigh molecular weight polyethylene
NS: Non-surgical
MD: Mean difference
TE: Treatment effect
SD: Standard deviation
CI: 95% Confidence interval
