# Supplementary material for: Treatment of post-meniscectomy knee symptoms with medial meniscus replacement results in greater pain reduction and functional improvement than non-surgical care
Source: Knee Surg Sports Traumatol Arthrosc. 2021 Apr 21;30(4):1325–35. doi: 10.1007/s00167-021-06573-0 (PMC9007779; doi:10.1007/s00167-021-06573-0)
Supplement: Supplementary file 1 — Supplementary file1 (DOCX 20 KB) [file 167_2021_6573_MOESM1_ESM.docx]

**Title:** Treatment of post-meniscectomy knee symptoms with medial meniscus replacement results in greater pain reduction and functional improvement than non-surgical care: 1-year results from the VENUS randomized controlled trial

**Journal:** Knee Surgery, Sports Traumatology, Arthroscopy (KSSTA)

| **Additional File 1. Mean scores and treatment effect for KOOS, VAS Pain, WOMET, and IKDC^a^** | | | | | | | | | | | | | |
| --- | --- | --- | --- | --- | --- | --- | --- | --- | --- | --- | --- | --- | --- |
|  | **1.5 Months** | | | |  | **6 Months** | | |  | **1 Year** | | |  |
|  | NS  (SD)  n=64 | | MMR  (SD)  n=61 | TE  (95% CI) |  | NS  (SD)  n=56 | MMR  (SD)  n=61 | TE  (95% CI) |  | NS  (SD)  n=46 | MMR  (SD)  n=57 | TE  (95% CI) |  |
| KOOS Pain^b^ | 62.0  (14.2) | 55.4  (18.4) | | -6.6  (-14.1 to 0.9) |  | 65.1  (17.5) | 74.3  (15.4) | 9.1*  (1.4 to 16.9) |  | 68.4  (18.3) | 78.0  (15.7) | 9.6*  (0.9 to 18.3) |  |
| KOOS Symptoms^b^ | 65.6  (16.5) | 45.4  (13.0) | | -20.1***  (-26.9 to  -13.4) |  | 66.8  (17.2) | 67.1  (16.8) | 0.3  (-7.7 to 8.2) |  | 70.8  (19.1) | 72.8  (18.4) | 2.0  (-7.5 to 11.5) |  |
| KOOS ADL^b^ | 72.0  (16.6) | 63.6  (16.6) | | -8.4*  (-16.0 to  -0.9) |  | 75.5  (20.3) | 82.8  (13.6) | 7.3  (-0.9 to 15.4) |  | 76.9  (18.8) | 85.7  (15.2) | 8.9*  (0.2 to 17.6) |  |
| KOOS Sports^b^ | 41.7  (27.8) | 28.7  (30.4) | | -13.0  (-26.3 to 0.3) |  | 46.3  (28.7) | 57.9  (24.6) | 11.6  (-1.0 to 24.1) |  | 52.3  (28.3) | 59.5  (28.6) | 7.2  (-7.1 to 21.5) |  |
| KOOS QOL^b^ | 35.1  (17.5) | 34.1  (20.1) | | -1.0  (-9.6 to 7.6) |  | 39.0  (21.4) | 53.6  (21.0) | 14.6**  (4.7 to 24.6) |  | 44.9  (22.5) | 55.8  (25.4) | 10.9  (-1.1 to 22.9) |  |
| KOOS Overall^b^ | 55.3  (14.9) | 45.2  (15.8) | | -10.1**  (-17.1 to -3.0) |  | 58.6  (17.6) | 67.1  (15.4) | 8.6*  (0.8 to 16.3) |  | 62.7  (18.6) | 70.4  (18.9) | 7.7  (-1.7 to 17.1) |  |
| VAS  Pain^c^ | 43.2  (25.6) | 37.5  (24.0) | | -5.7  (-16.9 to 5.6) |  | 37.8  (26.6) | 17.3  (16.0) | -20.5***  (-31.0 to -10.0) |  | 37.0  (28.4) | 20.0  (23.0) | -17.0**  (-30.1 to -3.8) |  |
| WOMET^b^ | 45.9  (21.2) | 45.0  (21.3) | | -1.0  (-10.6 to 8.7) |  | 48.6  (24.8) | 65.8  (21.6) | 17.2***  (6.2 to 28.1) |  | 54.1  (24.6) | 66.9  (25.4) | 12.7*  (0.2 to 25.3) |  |
| IKDC^b^ | — | — | | — |  | 52.5 (18.6) | 59.2  (17.1) | 6.8  (-1.2 to 14.8) |  | 54.7 (18.7) | 62.2  (20.8) | 7.5  (-2.1 to 17.0) |  |
| Abbreviations: NS, Non-surgical; MMR, Medial meniscus replacement; SD, Standard deviation; TE, Treatment effect; 95% CI, 95% confidence interval.  ^a^Means, treatments effects, and confidence intervals are derived from mixed-effects models with Sidak’s multiple comparisons tests.  **P*<0.05, ***P*<0.01, ****P*<0.001  ^b^ Higher values indicate less severe symptoms.  ^c^ Lower values indicate less severe symptoms. | | | | | | | | | | | | | |
